# Comprehensive Management of Ectodermal Dysplasia with Interceptive Orthodontics in a Young Boy Who Was Bullied at School

**DOI:** 10.1155/2020/6691235

**Published:** 2020-12-30

**Authors:** A. A. A. K. Wimalarathna, W. B. M. C. R. D. Weerasekara, E. M. U. C. K. Herath

**Affiliations:** ^1^Department of Prosthetic Dentistry, Faculty of Dental Sciences, University of Peradeniya, Sri Lanka; ^2^Department of Community Dental Health, Faculty of Dental Sciences, University of Peradeniya, Sri Lanka

## Abstract

**Aim:**

The management of hypohidrotic ectodermal dysplasia with oligodontia in Class-I malocclusion in late mix dentition. *Case Report*. An 11-year-old boy with ED was treated and managed by means of interceptive orthodontic treatment accompanied by direct and indirect restorative methods in a successful manner. The patient was prepared to receive definitive oral rehabilitation with dental implants for the missing teeth when the patient reaches a suitable age. The patient was followed for 5 years from the beginning of treatment.

**Conclusion:**

Management of the child with ectodermal dysplasia with oligodontia was a real challenge. Early diagnosis, necessary investigation, and providing age-appropriate multidisciplinary treatment were key steps in successful outcomes. The objectives were not only just orthodontic or paedodontics but also prosthetic and psychological.

## 1. Introduction

The term ectodermal dysplasia (ED) is described as a rare heterogeneous group of congenital disorders which is characterized by defects of two or more ectodermaly derived tissues [1]. These are primarily the nails, skin, hair, sweat glands, and teeth [2]. Though only eight forms of ED were identified by 1971, currently, it comprises more than 200 different types; about 30% have been identified at the molecular level with identification of the causative genes [3].

According to the mode of inheritance, ED can be classified into autosomal dominant, autosomal recessive, or X-linked. Hypodontia, hypohidrosis, and hypotrichosis are a triad of clinical features characteristic of the hypohidrotic form [4]. Based on the function and number of sweat glands, ED can be broadly divided into two clinically distinguished forms: first, autosomal dominant hidrotic type (Clouston's syndrome) with normal sweat glands, and second, the X-linked recessive anhidrotic or hypohidrotic type (Christ-Siemens-Touraine Syndrome) with absent or diminished sweat glands. The X-linked trait is the most common phenotype with an incidence of 1-7 per 100,000 live births, with 28% of mortality rate in males up to 3 years of age [3].

Patients with ED can easily be recognized from normal individuals by their typical facial and clinical features such as frontal bossing, prominent supraorbital ridges, sunken cheeks, wrinkles and periorbital hyperpigmentation, saddle nose, everted lips, large low-set ears, dysplastic nails, and reduced lower facial height [2]. Occasionally some of them have asthma and eczema [5].

Dental manifestations include multiple congenitally missing teeth, conical- or pegged-shaped anterior teeth, ankylosed primary molars due to missing permanents, delayed eruption of permanent teeth, hypoplastic alveoli, xerostomia and thickened frena, macrodontia or microdontia, enamel hypoplasia, and taurodontism [2]. Normally, children with ED have normal psychological and social development [6].

Considering oral aesthetics, rehabilitation of a patient with ED has a big challenge due to missing teeth. According to the number of missing teeth, it can be classified into hypodontia (missing less than six teeth), oligodontia (six or more missing teeth), and anodontia (completely absence of teeth) [7]. The problems associated with missing teeth are directly proportionate to the number of missing teeth in both deciduous and permanent dentition. The most rarely missing teeth are maxillary central incisors, maxillary canines, and maxillary first molars [8]. Mostly, the presenting teeth are conical in shape, malformed, with restricted alveolar bone development, and often showing unaesthetic spacing.

A multidisciplinary approach is now recommended for managing ED patients. Early and extensive dental treatment is needed throughout childhood because of the absence of most of the deciduous and permanent teeth, and they are still in the developing stage. Therefore, the management basically consists of prevention of caries, restoration of malformed teeth, replacement of missing teeth, management of infraocclusion, and correction of malocclusions [7].

Therefore, in many cases of hypodontia, orthodontic treatment can greatly facilitate any restorative treatment or sometimes even eliminate the need for it [9]. Carrying out orthodontic treatment in order to eliminate or to simplify later orthodontic treatment in young patients is called “interceptive orthodontics” (IO) [10]. The main aims of IO treatments in hypodontia are management of available space, uprighting and alignment of teeth, and management of increased overbite, as well as interdisciplinary inputs of hypodontia management [9].

The presenting case shows the total care management of a paediatric patient with ED which is associated with oligodontia was managed by the means of interceptive orthodontic treatment accompanied by direct and indirect restorative methods in a successful manner.

## 2. Case Report

An 11-year-old schoolboy presented to the paedodontic clinic with the main complaint of having abnormal and uneven teeth and requesting treatments. The parents had noticed that the permanent teeth were abnormal in shape and malaligned. Most importantly, when the child was inquired about his concern, he said that his biggest problem was being teased and bullied by his peers at school.

**Figure 1:**
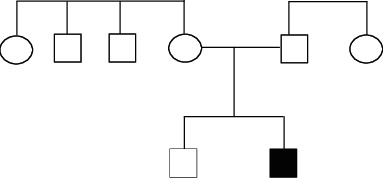
The genealogy of the presenting patient.

There was no history of trauma related to the teeth. The child has had a history of asthma but is otherwise healthy. He had been brushing his teeth with fluoridated toothpaste twice daily, using a toothbrush. The child's dietary habits did not show a high consumption of sugar. There was no history of parafunctional habits. Parents have no consanguineous marriage (Figure 1).

**Figure 2:**
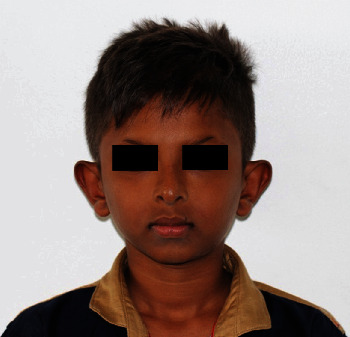
Facial image of the patient.

On examination, it was observed that the overall development was appropriate for his chronological age. On extraoral examination, he was noticed to have fine sparse hair, scanty eyelashes and eyebrows, protruding lips with reduced lower facial height, periorbital pigmentation, and protruding low set of ears (Figure 2). His palms and soles showed hyperkeratosis.

**Figure 3:**
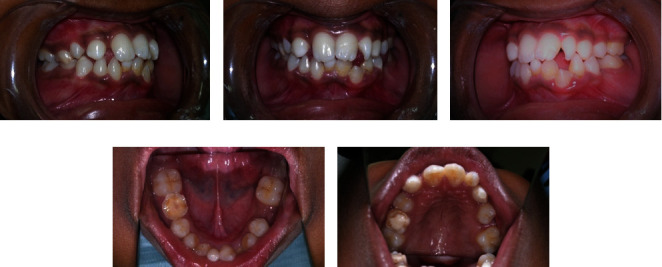
Preoperative intraoral images of (a) right buccal, (b) closed mouth, (c) left buccal, (d) lower arch, and (e) upper arch views.

Intraoral examination showed normal soft tissues and no signs of periodontal disease. The lower anterior teeth were stained and showed crowding with overretained 81 and 62. There was a superficial dentinal caries on 73 with slight mobility. The lower permanent incisors (32 and 42) were conical in shape (Figure 3).

All four deciduous canines were slightly overretained with the canine bulges evident in relation to 13 and 43, whereas 33 had already erupted. There was a restoration on 55 with secondary caries. With mild attrited facets on 85, the occlusal surface showed slight mobility. 65 and 75 had been extracted due to caries, and 37 was erupting (Figures 3). His *dmft* index was 4. He had a Class-I skeletal pattern, with average “Frankfort mandibular plane angle” associated with incisor Class-I and bilateral molar Class-II 1/2 unit relationship. Lips were competent and everted. There was evidence of normal tongue and adaptive tongue thrust during the activity.

**Figure 4:**
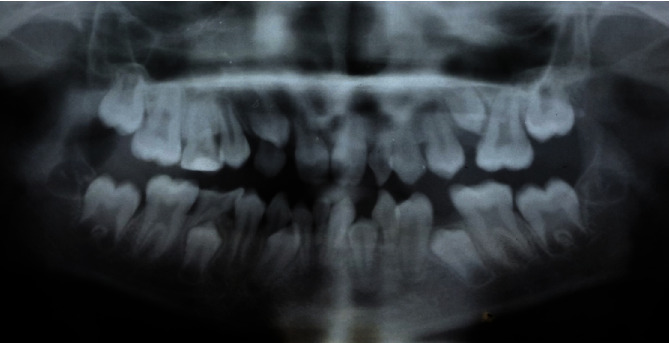
Preoperative photoimage of dental panoramic tomograph.

The radiographic investigation, sensibility tests, and basic periodontal examination (BPE) were performed to identify missing permanent teeth, pulp status, and periodontal health, respectively. All the teeth gave a vital response, and the plaque score was 29%. There was no evidence of deep pockets more than 3 mm. The radiological findings revealed 6 missing teeth: 12, 15, 22, 23, 31, and 41 while 25 was impacted due to space loss. There were anterior conical teeth and overretained 81 with resorbing root and taurodontic molar teeth (Figure 4).

With the information gathered from the history, examination, and investigations, this case was diagnosed as “a patient with hypohidrotic-type ectodermal dysplasia with oligodontia in Class-I malocclusion in late mix dentition on skeletal base Class-I.”.

Written consent was obtained from the parents after explaining the proposed treatment plan. At the initial stage, the routine preventive measures like the use of fluoridated toothpaste and correct brush instructions were introduced after plaque demonstration. At the second visit, dietary modification and counselling were done. Fluoride varnish was introduced for professional application. Further CPP-ACP containing GC tooth mousse plus was introduced to use at home on a daily basis.

**Figure 5:**
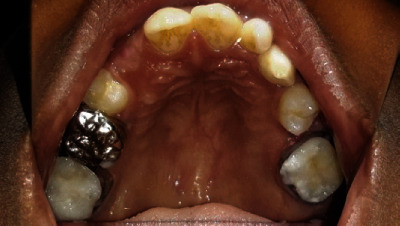
Intraoral photograph shows cemented crown and molar bands.

During the third visit, the child demonstrated a good relationship with the dental team and seemed to have adapted well to the clinical environment. According to the treatment plan, 53, 73, and 83 were extracted after placing separators to facilitate molar bands and stainless steel crown on 55 in the next visit. At the fourth visit, fissure sealants on permanent molars and cementation of a stainless steel crown for 55 were done using luting GIC. The molar bands were cemented to all first molars (Figure 5).

During that period, the permanent canines erupted as expected after extraction of deciduous canines; the patient was reassessed at the ortho-paedodo joint clinic, and the following management steps were planned: derotation of 13 and 24, surgical exposure of 25, extraction of 81, uprighting and distalization of distally tilted 72, distalization of 26 to facilitate the eruption of 25, recontouring of upper and lower incisors with composites after completing orthodontic treatment, and replacement of missing 31 and 41 spaces with one tooth with a cantilevered resin-bonded bridge (RBB) from 32.

**Figure 6:**
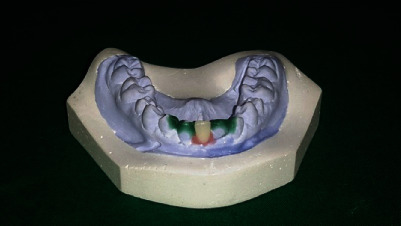
Lower arch “Kesling diagnostic setup.”

**Figure 7:**
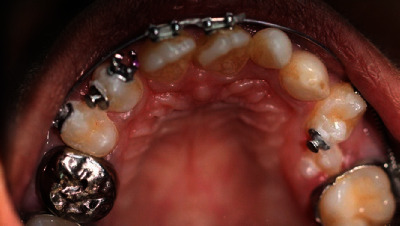
Intraoral photograph showing cemented palatal button on rotated 13 and 24.

**Figure 8:**
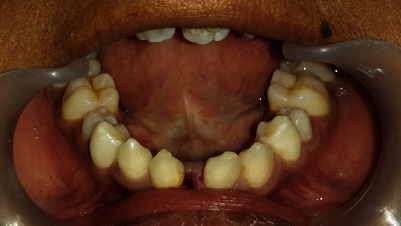
Intraoral photograph showing immediately after extraction of 81.

Before commencing the second half of the orthodontic treatment, the lower arch “Kesling diagnostic setup” was made (Figure 6). At the next visit, palatal buttons were fixed on rotated 13 and 24 in order to derotate (Figure 7). The overretained 81 was extracted prior to bond up of the lower arch (Figure 8).

**Figure 9:**
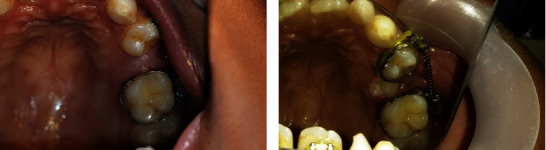
Intraoral photographs of 25 (a) before and (b) after surgical exposure.

**Figure 10:**
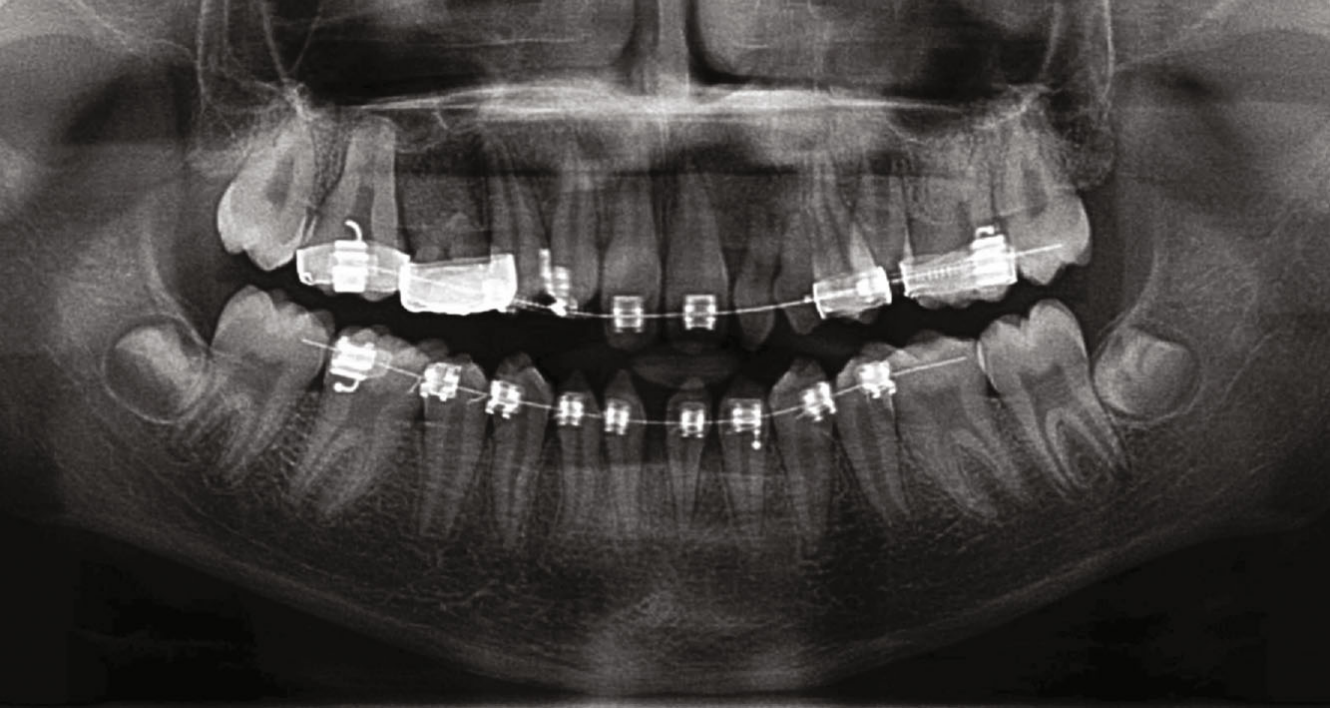
The “near-end radiograph” (digital dental panoramic tomograph).

In parallel to the orthodontic treatment, surgical exposure of 25 and distalization of 26 with a coiled spring was done to facilitate the eruption of 25 (Figure 9). Prior to debonding the fixed applications, a “near-end radiograph” was taken to observe the status of root resorption, teeth position, and the available spaces and root parallelism for future implant placement of the overretained teeth (Figure 10).

**Figure 11:**
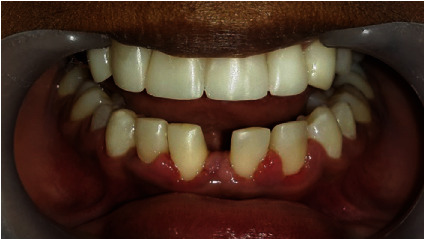
All conical incisors were recontoured with LCC.

**Figure 12:**
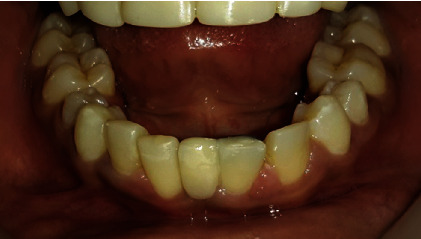
RBB was cemented to replace missing lower central incisors.

Immediately after debonding, all the conical incisors and canines were recontoured using composites (Figure 11). The missing upper laterals were managed by right-side canine lateralization and left-side overretained deciduous canine recontouring by selective grinding and composite buildups. The missing 41 was replaced by a cantilever RBB (Figure 12).

**Figure 13:**
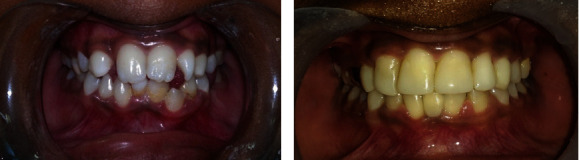
Comparison of preoperative and postoperative aesthetic outcomes.

The total care rehabilitation, including aesthetic and preventive measures, seemed to be alright in our patient at the end of the 5-year follow-up period (Figure 13). He is still on review. Implant-supported crowns for the missing 15, 22, 23, 31, and 41 could be considered when the patient is at the age of 18 years.

## 3. Discussion

Ectodermal dysplasia associated with oligodontia is a complex dental condition that significantly effects on aesthetics and function. In order to provide aesthetically and functionally pleasing outcomes, a multidisciplinary management approach may be required. The aims of that approach should include improving aesthetics, maintaining the existing dentition, improving speech, enhancing the masticatory efficacy, improving acceptance by family and peers, and promoting psychological wellbeing [7]. Most of these requirements can be achieved by addressing the hypodontia by replacing missing teeth with recommended options such as removable prosthesis, conventional or resin adhesive bridgework, implant-supported prosthesis, or autotransplantation [11]. All the options have a common prerequisite in which they need space management before replacing the missing teeth.

Therefore, managing the child especially in mixed dentition with ED and interceptive orthodontics plays a major role. The patient is awaiting the orthodontic treatments which mean he/she became a candidate for the preventive care like plaque control, fissure sealant, dietary counselling, and fluoride therapy [12]. Therefore, our patient also underwent all the preventive measures. Even though we clinically diagnosed this case as hypohidrotic ED, the ideal way of confirming it is a skin biopsy that shows absent or hypoplastic sweat glands. Furthermore, to diagnose the subtypes of EDs, many ways of genetic tests are also available [13].

Currently, there is no causative treatment available [14]. ED-associated hypodontia can be affected by both deciduous and permanent dentition [15]. Most often, the permanent maxillary central incisors, maxillary first molars, mandibular first molars, and maxillary canines are present in hypohidrotic ED [16]. The most common concern of the children with ED is about the dental anomalies and facial appearance [17], and it was common for the present case study also.

The main demand of these patients is replacing their missing teeth and recontouring of the malformed teeth to improve their aesthetics and functions. But the conventional prosthodontic treatments are considered problematic in severe hypodontic cases [18] that may be associated with underdeveloped alveolar ridges in edentulous areas and malformed or overretained deciduous abutment teeth which will lead to poor support, stability, and retention of the prosthesis.

The new trends of managing EDs are moving towards implant-supported rehabilitation [19]. Therefore, the introduction of interceptive orthodontics to the ED patients is of utmost importance to prepare them as candidates for future implant therapy. Interceptive orthodontic treatments are a method to restore a normal occlusion when a malocclusion has started to occur. Furthermore, the development of the dentoskeletal complex, possible discrepancies, and malposition are identified and removed throughout the interceptive orthodontic period. Applications undertaken in interceptive orthodontics are serial extraction, correction of developing crossbite, control of abnormal habits, space reclamation and distribution, extraction of supernumerary and retained primary teeth, root parallelism, and correction of possible predictable malocclusions [20, 21]. The main factors guiding the decision towards the orthodontic and prosthetic choice are the presence of posterior natural teeth, facial aesthetics, lip support, number and size of existing natural teeth, and the occlusal vertical dimension.

The successful outcomes of the patient presented in this case report are due to good understanding and the commitments of both clinicians and the child's parents, who always had positive attitudes towards the proposed multidisciplinary treatment plan.

## 4. Conclusion

Management of a child with ectodermal dysplasia with oligodontia was a real challenge. Early diagnosis, necessary investigation, and providing age-appropriate multidisciplinary treatment were key steps in successful outcomes. Furthermore, selecting the most suitable treatment option among a verity of modalities is of utmost importance to the long-term sustainability of both provided and future treatments as well. Finally, providing the treatments for ED patients while preparing them for future implant therapy is an added advantage for the patients. Most importantly, upliftment of the psychological wellbeing of the young children provided by proper management of ED is of utmost importance to improve their quality of life.

## Data Availability

All the available data which relevant to this case report has presented in the manuscript.
